# Phospholipase Cδ-4 (PLCδ4) Acts as a Nuclear Player to Influence Cyclin B Expression in the Embryonal Rhabdomyosarcoma Cell Lines RD and A204

**DOI:** 10.3390/biom14091180

**Published:** 2024-09-20

**Authors:** Sara Salucci, Alberto Bavelloni, Ilaria Versari, Sabrina Burattini, Francesco Bavelloni, Pietro Gobbi, Alessandro Fanzani, Silvia Codenotti, William Blalock, Katia Scotlandi, Irene Faenza

**Affiliations:** 1 Department of Biomedical and NeuroMotor Sciences (DIBINEM), University of Bologna, 40126 Bologna, Italy; ilaria.versari4@unibo.it (I.V.); francesco.bavelloni25@gmail.com (F.B.); 2 Laboratory of Experimental Oncology, IRCCS, Istituto Ortopedico Rizzoli, 40136 Bologna, Italy; alberto.bavelloni@ior.it (A.B.); katia.scotlandi@ior.it (K.S.); 3 Department of Biomolecular Sciences (DISB), Urbino University Carlo Bo, 61029 Urbino, Italy; sabrina.burattini@uniurb.it (S.B.); pietro.gobbi@uniurb.it (P.G.); 4 Department of Molecular and Translational Medicine, University of Brescia, 25123 Brescia, Italy; alessandro.fanzani@unibs.it (A.F.); silvia.codenotti@unibs.it (S.C.); 5 IRCCS, Istituto Ortopedico Rizzoli, 40136 Bologna, Italy; william.blalock@cnr.it; 6 “Luigi Luca Cavalli-Sforza” Istituto di Genetica Molecolare, Consiglio Nazionale delle Ricerca (IGM-CNR), 40136 Bologna, Italy

**Keywords:** human rhabdomyosarcoma, PLCδ4, cyclin B1, Akt, immunolocalization, proteome array

## Abstract

Rhabdomyosarcoma (RMS), the most common form of sarcoma typical of pediatric age, arises from the malignant transformation of the mesenchymal precursors that fail to differentiate into skeletal muscle cells. Here, we investigated whether the protein phospholipase C δ4 (PLCδ4), a member of the PLC family involved in proliferation and senescence mechanisms of mesenchymal stromal stem cells, may play a role in RMS. Our molecular and morpho-functional data reveal that PLCδ4 is highly expressed in the fusion-negative, p53-positive, SMARCB1 heterozygous mutated embryonal RMS (ERMS) cell line A204, while it is poorly expressed in the ERMS cell lines RD (fusion-negative, MYC amplification, N-RAS (Q61H), homozygous mutated p53) and Hs729 (homozygous mutated p53) and the alveolar rhabdosarcoma (ARMS) cell line SJCRH30 (RH30; fusion positive, heterozygous mutated RARA, polyheterozygous mutated p53). To characterize the role of PLCδ4, the RD cell line was stably transfected with wild-type PLCδ4 (RD/PLCδ4). Overexpressed PLCδ4 mainly localized to the nucleus in RD cells and contributed to the phosphorylation of PRAS40 (T246), Chk2(T68), WNK1(T60), and Akt 1/273 (S473), as revealed by proteome profiler array analysis. Overexpression of PLCδ4 in RD cells enhanced cyclin B1 expression and resulted in G2/M-phase cell cycle arrest. In contrast, siRNA-mediated knockdown of PLCδ4 in A204 cells resulted in reduced cyclin B1 expression. Our study identifies a novel role for nuclear PLCδ4 as a regulator of cyclin B1 via Akt-dependent phosphorylation. The modulation of PLCδ4 expression and its downstream targets could represent a crucial signaling pathway to block embryonal RMS cell proliferation.

## 1. Introduction

In mammals, the phospholipase C (PLC) family includes 13 isozymes divided into six classes based on their structure: β, δ, γ, ε, ζ, η [1,2,3,4].

PLC catalyze the reaction of phosphatidylinositol 4,5-bisphosphate (PI(4,5)P2) cleavage to generate two second messengers, inositol 1,4,5 triphosphate (IP3) and di-acylglycerol (DAG), which, respectively, regulate intracellular Ca^2+^ levels and the activation of protein kinase C. All these isozymes, which may differ in tissue distribution, intracellular location, regulatory mechanism, and have crucial roles in signal transduction, are linked to human disease development [5]. Moreover, PLC family enzymes are correlated with several diseases, including cancer [6], schizophrenia [7], epileptic encephalopathy [8], and myotonic dystrophy [9,10] and in hematopoietic stem cell proliferation and differentiation [11]. It has been well demonstrated that PIP2, like PLC isoforms β1, δ1, and δ4, is present in the nucleus, where it contributes to cell proliferation, cell cycle regulation, differentiation, membrane trafficking, and gene expression [1,2,12,13,14,15].

PLCδ4 has been described as a nuclear protein in multiple cell types and tissues. The nuclear expression of PLCδ4 is most enhanced in growing cells and is induced during the S-phase [16]. Despite its nuclear localization, the mechanism of activation of PLCδ4 in the nucleus is not yet understood. The human homolog of PLCδ4 has not been widely characterized, but it could be associated with pathologies and cellular mechanisms like those described for other PLC isoforms, such as cancer development and neurodegenerative diseases. In SKHep-1, a human liver cancer cell model, and in human hepatocytes, PLCδ4 is localized within the nucleus and participates in cell cycle progression downstream of EGFR, by reducing the expression of cyclins A and B1 [17]. 

Recent data from Kunrath-Lima et al. [18] showed that human PLCδ4 (PLCδ4), localized in the nucleus, is involved in mesenchymal stromal stem cell proliferation and the modulation of their senescence. In fact, PLCδ4 knockdown promoted cell cycle arrest without influencing cell death, thus increasing the percentage of senescent cells and the expression of p16INK4A+ and p21Cip1 mRNAs. Accordingly, we investigated the role of PLCδ4 in cell signaling and proliferation in RMS, the most common soft tissue sarcomas (STSs) in children. RMS is a highly aggressive form of sarcoma that develops from embryonic mesenchymal stem cells (EMSs) that have failed to fully differentiate into myocytes of the skeletal muscle. It can occur in all types of muscle tissue in any location, resulting in a high variability of clinical manifestations. Because RMS is a cancer originating from very early forms of muscle cells, it is much more common in children, although RMS does occur in adults [19,20]. Pathologists divide rhabdomyosarcoma into four types based on the appearance of the cells when examined under a microscope and on molecular changes within the tumor: embryonal (ERMS) and alveolar (ARMS), which are the most common types of RMS; spindle cell/sclerosing; and pleomorphic. ERMS usually affects children in their first 5 years of life, but it can occur at older ages as well. ARMS typically affects all age groups equally, and it makes up a larger portion of RMS in older children, teens, and adults as compared to RMS in younger children [21]. Nevertheless, with the advancement of molecular techniques, the classification of RMS has been expanding within the last decade. Treatment of RMS is a multidisciplinary practice involving the use of surgery, chemotherapy, radiation, and possibly immunotherapy [22]. Previous studies examining the antitumor mechanism of curcumin in RMS indicated that the PI3K/AKT/mTOR pathway, which is regulated downstream of PLC enzymes, is highly important for RMS maintenance [23]. 

In this study, we investigated the role of PLCδ4 in the ERMS cell line RD and in a poorly differentiated RMS cell line A204. We identified for the first time that cyclin B1 is a phospho-substrate of Akt in cells in which overexpression of PLCδ4 in the nucleus is observed.

## 2. Materials and Methods

### 2.1. Cell Culture and Reagent

RMS cell lines used in this study, A204, RD, Hs729, and SJCRH30; the osteosarcoma cell lines Saos, Hos, U2Os, and MG63; the Ewing cell line A673; and the liposarcoma cell lines T778 and SW872 were purchased from the American Type Culture Collection (ATCC; Manassas, VA, USA). Cell lines were routinely cultured in Dulbecco’s modified Eagle’s medium supplemented with 10% inactivated fetal calf serum (FCS; Life Technologies Corporation, Monza, Italy) and 2 mM L-glutamine (Sigma-Aldrich, St. Louis, MO, USA). Cells were maintained at 37 °C in a humidified 5% CO_2_ atmosphere. Vincristine and ixabepilone were purchased from Sigma-Aldrich. MK-2206 was purchased from Sellekchem, GmbH, 50829 Cologne, Germany.

### 2.2. Antibody Arrays

Cells were collected and lysed in kit-supplied lysis buffer and pre-cleared of cellular debris by centrifugation. Protein lysates were then quantitated using the Bio-Rad BSA Protein Reagent. For each antibody array, 500 µg of cellular extract was incubated with the Phospho-Kinase Array Kit membrane (Proteome Profiler; R&D Systems, Abingdon, UK), according to the manufacturer’s instructions. Following incubation with antibody and streptavidin–HRP conjugate, filter images were acquired on a Bio-Rad ChemiDoc XRS system (Bio-Rad, Segrate, Italy). Densitometry values of spots were estimated by ImageJ software and expressed as arbitrary units. Multiple film exposures were used to verify the linearity of the samples analyzed and to avoid saturation of the film. In antibody arrays, the average signal of the pair of duplicate spots, representing each phosphorylated kinase protein, was calculated after the subtraction of background values (pixel density) from negative control spots, and normalization to average values from positive control spots.

### 2.3. Protein Extraction and Western Blotting

Cells were lysed in radioimmunoprecipitation assay (RIPA) lysis buffer containing the complete EDTA-free protease and phosphatase inhibitor cocktails (Pierce, Thermo Fisher Scientific) and 25 units/mL of the pan-nuclease, benzonase (Sigma-Aldrich). Cells were lysed by vortexing for 30 min at 4 °C and then cleared of cellular debris by centrifugation at 14,000× *g* for 15 min at 4 °C. The protein concentration of the cleared lysates was determined using the Bradford protein assay reagent (Bio-Rad, Segrate, Italy). Proteins were separated by sodium dodecyl sulfate polyacrylamide gel electrophoresis (SDS-PAGE) on 4–20% gradient gels and immunoblotted. Primary antibodies used were as follows: Cell Signaling Technologies (Beverly, MA, USA): anti-Akt (pan) (Cat #4691; 1:1000), anti-phospho-Ser473 Akt (Cat #4060; 1:1000), anti-phospho-Akt substrate (RXXS*/T*) (Cat #9614; 1:1000), anti-phospho-PRAS40 (Thr246) (Cat #2997; 1:1000). Santa Cruz Biotechnology (Dallas, TX, USA): anti-PLCdelta4 (sc-373875; 1:1000), anti-cyclin A (sc-596; 1:1000), anti-cyclin B1 (sc-752; 1:1000). Sigma-Aldrich (Saint Louis, MI, USA): anti-β-tubulin (Sigma-Aldrich; 1:10,000, anti-FLAG (Cat# F1804).

### 2.4. Cell Proliferation

RD and RD/PLCδ4 cells were plated in 6-well tissue culture plates at a density of 2 × 10^5^ cells/well for 24, 48, 72, and 96 h. Cells in the respective wells were washed with 1× PBS, and resuspended in a buffer solution of 1× PBS, 0.5 mM EDTA and 0.2% BSA. Then, 50 µL of cell suspension was mixed with an equal volume of 0.4% Trypan blue. The solution was thoroughly mixed and allowed to stand for 5 min at room temperature. Then, 10 µL of the solution was pipetted into a disposable chamber slide and inserted into an automated cell counter (Countess™, Thermo Fisher Scientific).

### 2.5. MTT Assay

Cells were seeded into 96-well plates at a density of 5000 cells/well, in 100 µL of cell culture medium, and incubated for 24 h to allow cell adherence. To test the effects of vincristine or ixabepilone, growth media were exchanged, and the cells were cultured for 24, 48, or 72 h, either in the presence of the vehicle (DMSO 0.1%) or increasing concentrations of both drugs (10 pM–10 nM). Cell growth was determined using resazurin-based PrestoBlue reagent (Invitrogen, Monza, Italy), according to the manufacturer’s instructions. Briefly, the PrestoBlue solution (10 µL) was added to each well, and the plates were then incubated for an additional 2 h. The plates were then directly read on an Infinite M200 photometer (Tecan Group Ltd., Mannedorf, Switzerland) at a wavelength of 600 nm.

### 2.6. Cell Cycle Analysis

To analyze the cell cycle distribution, RD and RD/PLCδ4 cells were grown for 4, 24, and 48 h. Cells were collected, washed once with ice-cold PBS 1×, and fixed in ice-cold 70% ethanol at −20 °C for 24 h. The fixed cells were centrifuged at 1500× rpm for 5 min at 4 °C; then, the cell pellet was washed twice with ice-cold PBS and stained with 0.5 mL FxCycle™ PI/RNase Staining Solution. The cell cycle distribution was evaluated from 10,000 counts using an Attune Nxt Acoustic Focusing Cytometer, equipped with a blue laser (488 nm) (Life Technologies Corporation, Monza, Italy). Data were acquired in list mode using Attune Cytometric 2.6 software. 

### 2.7. Immunofluorescence, Immunohistochemistry, and Confocal Laser Scanning Microscopy (CLSM)

Cells, grown on coverslips, were fixed in 4% paraformaldehyde in 0.1 M phosphate-buffered saline (PBS), pH 7.4 for 30 min, and permeabilized with 0.20% Triton X-100 in PBS, for 10 min at RT. Then, samples were rinsed again with PBS, treated with 2% bovine serum albumin (BSA) and 5% normal goat serum (NGS) in PBS (PBS-NGS-BSA mixture), for 30 min at RT, and incubated with mouse or rabbit primary antibodies against cyclin B1 and Akt, respectively (1:200 in the PBS-NGS-BSA mixture), overnight at 4 °C. The next day, samples were rinsed with PBS and incubated with an FITC-conjugated goat anti-rabbit secondary antibody (Millipore, Burlington, MA, USA; 1:50 in the PBS-BSA-NGS mixture), and with a CY3-conjugated goat anti-mouse secondary antibody (Millipore; 1:50 in the PBS-BSA-NGS mixture), for 1 h at RT in the dark. 

For PLCδ4 immunolocalization, cells grown on coverslips and fixed in 4% paraformaldehyde in 0.1 M phosphate-buffered saline were permeabilized with 0.20% Triton X-100 in PBS, for 10 min at RT, and rinsed again with PBS. Samples treated with 2% BSA and 5% normal horse serum (NHS) in PBS (PBS-NHS-BSA mixture), for 30 min at RT, were incubated with a mouse monoclonal primary antibody (1:100) overnight. The next day, after PBS washing, cells were incubated with a FITC-conjugated horse anti-mouse secondary antibody (Millipore; 1:50 in the PBS-BSA-NHS) for 1 h at RT in the dark

Control sections for non-specific staining were treated with the same incubation protocol without the primary antibody. Slices were finally mounted with the Vectashield mounting media (Vectorlabs, Newark, CA, USA). 

Images were analyzed by means of a Leica TCS-SP5 confocal microscope, connected to a DMI 6000 CS inverted microscope (Leica Microsystems CMS GmbH, 35578 Wetzlar Germany) and analyzed using the software Leica Application Suite Advanced Fluorescence (LAS AF Version 2012.1.1). Samples were examined using an oil immersion objective lens (40× N.A. 1.25). Excitation was at 488 nm (FITC) and 543 nm (CY3); emission signals were detected at 519 nm and 598 nm, respectively. CLSM images are presented as single-plane images, and image analysis was carried-out using ImageJ 1.54j software (National Institutes of Health) [20].

### 2.8. Immunoprecipitation

The lysates (1 mg) in RIPA buffer were precleared for 2 h at 4 °C with 20 µL of protein A/G-agarose (SCBT) and centrifuged. The supernatants were incubated at 4 °C overnight with primary antibody: anti-AKT1, anti-cyclin B1, anti-FLAG Agarose affinity gel (20 µL) (MERCK), and anti-phospho-(Ser/Thr) AKT substrate (R/KXR/KXXS/T*) (Cell Signaling Technology, Danvers, MA, USA). Each sample was incubated for 2 h with 20 µL of protein A/G-agarose at 4 °C with agitation. The immunoprecipitate was washed 4 times in washing buffer [20 mMTris-HCl (pH 7.4), 10% glycerol, and 1% Nonidet P-40]. Immune complexes were resuspended in 20 µL of Laemmli sample buffer and boiled for 5 min at 95 °C; the supernatant was then separated by SDS-PAGE. The proteins were transferred to nitrocellulose membrane and blotted with anti-AKT1, anti-cyclin B1, anti-PLC δ4, or anti-phospho-(Ser/Thr) AKT substrate.

### 2.9. Apoptosis Assay

Apoptosis was then evaluated by flow cytometry analysis using the Phycoerythrin Annexin V detection kit I (BD Biosciences, San Jose, CA, USA), exploiting the binding of PE-conjugated Annexin V for the detection of apoptotic and necrotic cells. Secondary staining with 7-amino-actinomycin D (7-AAD) allowed for the identification of early apoptotic cells. Cell staining was performed according to the manufacturer’s instructions. Fluorescence resulting from PE and 7-AAD was measured at 530 nm and 620 nm, respectively. Stained cells (10,000 cells/sample) were acquired on an Attune Nxt Acoustic Focusing Cytometer (Life Technologies), and data were analyzed using Attune Cytometric 2.6 software (Life Technologies).

### 2.10. Cell Transfections

PLCδ4 overexpression: RD cells were transfected with Flag-tagged human PLCδ4 cloned into pCMV6 plasmid (OriGene Technologies, Rockville, MD, USA). Stable transfection was performed at 90% confluence, using Lipofectamine 2000 Reagent (Invitrogen, Thermo Fisher Scientific, Monza, Italy) according to the manufacturer’s instructions. To obtain stable overexpression, cells were selected in Dulbecco’s modified Eagle’s medium supplemented with 10% inactivated FCS containing Geneticin (G418; Sigma Aldrich) at a concentration of 500 μg/mL 24 h after the transfection.

PLCδ4 silencing: the expression of PLCδ4 in A204 cells was knocked down by the siRNA interference technique. Validated PLCd4-siRNA (Silencer siRNA: s3946, s 39459, s39458) and negative-control siRNA were obtained from Invitrogen (Van Allen Way Carlsbad, CA, USA). A204 cells were seeded at a density of 2.0 × 10^4^ cells per well in 6-well plastic culture plates and cultured in opti-mem I reduced Serum Medium (GIBCO) for 24 and 48 h at 37 °C. A total of 30 nM of PLCδ4 siRNAs, and negative control siRNAs, were transfected into the cells using lipofectamine RNAiMAX reagent (Invitrogen), according to the manufacturer’s instructions. The above steps were repeated 24 h later and we transfected once more, and cells were collected 48 h later.

## 3. Results

### 3.1. PLCδ4 Is a Nuclear Protein in A204 Cells

**Figure 1:**
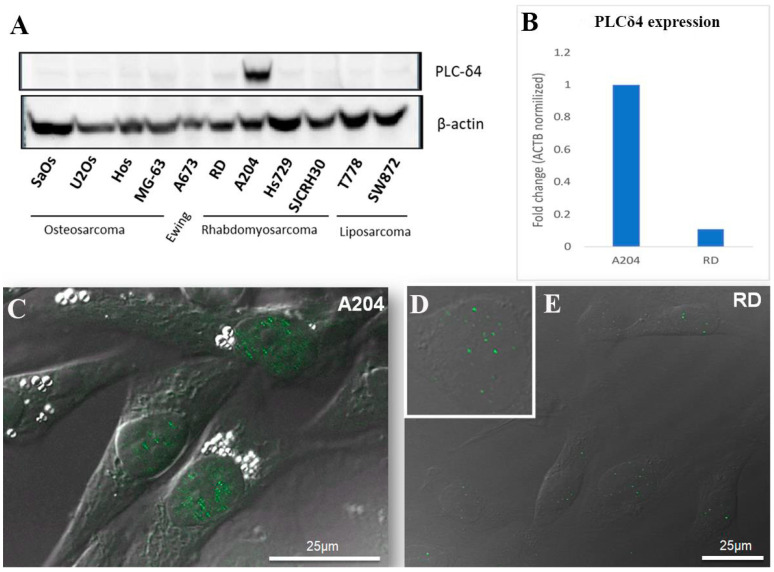
Expression of PLCδ4 in sarcoma cell lines (**A**). Total cell lysates (40 μg) were separated on 8% SDS-PAGE, transferred to nitrocellulose, and immunoblotted with the indicated antibodies. mRNA (**B**) expression and immunolocalization (**C**–**E**) of PLCδ4 in A204 and RD cells. Results represent the average of three independent experiments. All original figures of WB can be found in Supplementary Materials Files.

We investigated the expression of PLCδ4 in eleven human sarcoma cell lines: osteosarcoma (SaOS-2, U2OS, Hos, MG63), Ewing sarcoma (A673), liposarcoma (T778 and SW872), and RMS (RD, A204, Hs729, and SJCRH30). When comparisons were made, we found that PLCδ4 is expressed more intensely in the A204 RMS cell line, while it is only slightly detected in the RD RMS cell line and almost completely absent in the remaining sarcoma cells (Figure 1A). Expression was also assessed by qPCR. In qPCR assays, PLCδ4 gene expression was much higher in A204 compared to RD cells (Figure 1B). This difference may be attributable to the fact that A204 is a wild-type p53-expressing ERMS cell line, while the other tested RMS cell lines express either homozygous mutant p53 (RD and Hs739) or polyheterozygous mutated p53 (SJCRH30). As PLCδ4 is an enzyme that has been described to predominantly localize in the nucleus, to assess the localization of PLCδ4 in RMS cells, A204 and RD cells were assayed by immunofluorescent microscopy using an anti-PLCδ4 antibody. Data demonstrated that PLCδ4 is mainly expressed in the nuclei of A204 cells (Figure 1C); it is also detectable albeit with less intensity in RD cells (Figure 1D,E).

### 3.2. PLCδ4 Overexpression Causes G2/M Cell Cycle Arrest

**Figure 2:**
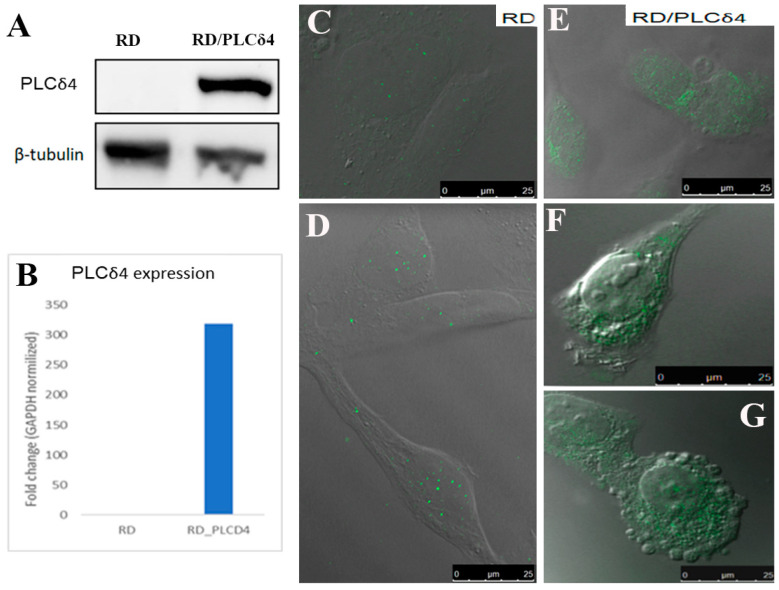
Expression and localization of PLCδ4 in RD cells and RD cells stably transfected with PLCδ4. (**A**) Whole homogenates of RD and RD/PLCδ4 were separated by SDS–PAGE and analyzed by immunoblotting with specific antibodies. (**B**) mRNA expression. Immunolocalization of PLCδ4 in RD cells (**C**,**D**) and in RD cells stably transfected with PLCδ4 (**E**–**G**). Results represent the average of three independent experiments.

To evaluate the role of PLCδ4, its stable overexpression was carried out using RD cells. Overexpression efficiency was assessed by Western blotting (Figure 2A,B) and qPCR. Protein and gene expression results confirmed that RD/PLCδ4 cells indeed overexpress PLCδ4 compared to control parental RD cells. Immunocytochemical analyses showed that in RD parental cells, a weak expression of PLCδ4 can occasionally be observed (Figure 2C,D). 

**Figure 3:**
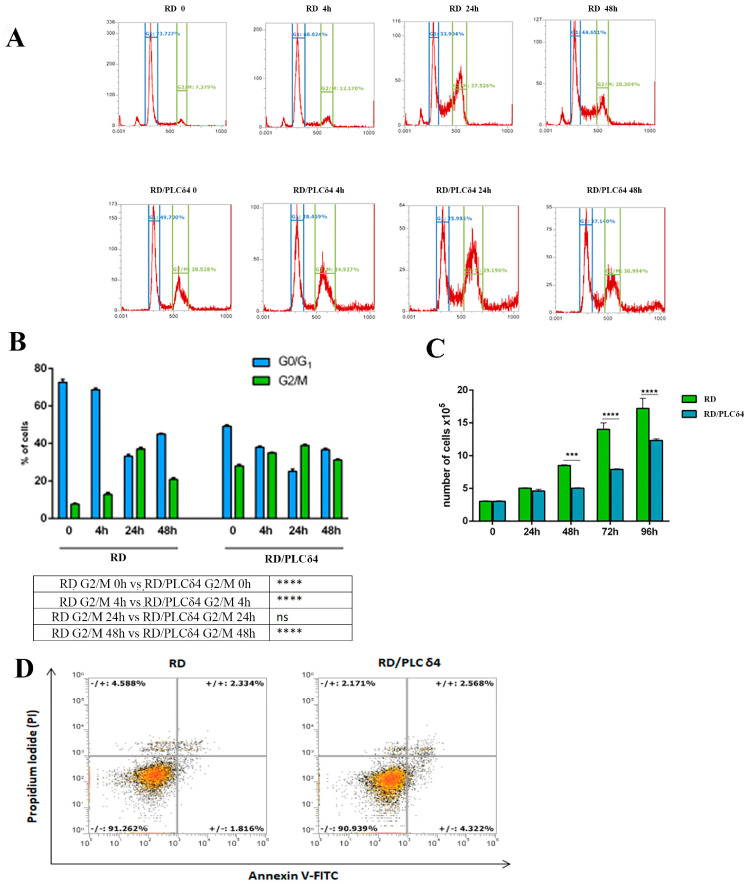
Overexpression of PLCδ4 in RD cells induces an arrest in the G2/M phase and a reduction in cell proliferation. (**A**) RD and RD/PLCδ4 cells were seeded and then analyzed at 4 h, 24 h, and 48 h by flow cytometry to determine the cell cycle distribution of the cells. (**B**) The graph shows the percent distribution of cells in each of the cell cycle phases and is representative of three independent experiments. **** *p* < 0.0001. (**C**) Histograms show the percentage of cells after 24, 48, 72, and 96 h and are representative of three independent experiments. *** *p* < 0.001; **** *p* < 0.0001. Statistical analyses were performed through two-way ANOVA. (**D**) Flow cytometry plots of cells stained with Annexin V-FITC/PI. Data are mean of three measurements. ns: not significant.

Overexpression of PLCδ4 in RD cells was investigated using CLSM, which evidenced its distribution inside the nucleus and its diffusion through the cytoplasm (Figure 2E–G). When PLCδ4 is upregulated, morphological changes appear, and some cells lose their fusiform and star-shaped aspect, becoming round. Moreover, the plasma membrane protrudes like a bubble due to its dissociation from the cytoskeletal network. In these cells, PLCδ4 localized among the membrane blebs and apoptotic bodies. Once the efficiency of PLCδ4 overexpression was confirmed, functional analyses were conducted with RD/PLCδ4 cells. Cell cycle (Figure 3A,B) and cell proliferation (Figure 3C) assays revealed that PLCδ4 expression resulted in a significant G2/M arrest with a reduced rate of cell division as compared with the parental RD cells. Furthermore, Annexin V-FITC/PI analysis (Figure 3D) did not reveal the presence of apoptosis in RD/PLCδ4 cells at the experimental times considered.

### 3.3. RD/PLCδ4 Cells Alter Diverse Signal Transduction Pathways Important to Tumor Growth

**Figure 4:**
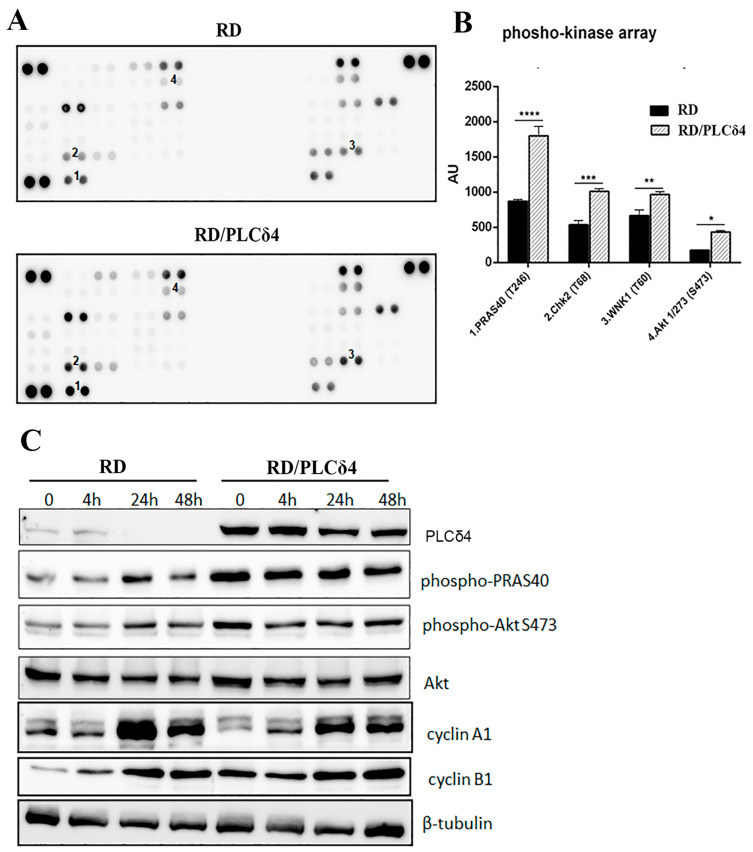
Phospho-kinase array analysis of RD and RD/PLCδ4. (**A**) Whole-cell lysates were prepared from RD and RD/PLCδ4 and hybridized with a Phospho-Kinase array kit. Spot **1**: phosphor-PRAS40, spot **2**: ChK2, spot **3**: WNK1, spot **4** phospho-Akr S473 (**B**) Spot densities of phospho-proteins were quantified using ImageJ software and normalized to those of positive controls on the same membrane. * *p*, 0.05 ** *p*, 0.01, *** *p*, 0.001, **** *p*, 0.0001. (**C**) RD and RD/PLCδ4 cells were seeded in a 6-well plate at a concentration of 2 × 10^5^ cells/well. After 4, 24, and 48 h, cells were collected and cell lysates (30 µg) were separated by 10% SDS-PAGE and immunoblotted with specific antibodies directed against: PLCδ4 (1:1000, overnight), pPRAS40 (1:1000, overnight), Akt (1:1000, overnight), *p*-Akt (S473) (1:1000, overnight), cyclin A1 (1:1000, overnight), cyclin B1 (1:1000, overnight), and β-tubulin (1:5000, 1 h). Results represent the average of three independent experiments.

To identify the underlying molecular mechanism of overexpression of PLCδ4 in RMS, we used the Proteome Profiler Human Phospho-Kinase Array Kit, a membrane-based sandwich immunoassay closely related to cell proliferation and migration. Phospho-kinase array analysis of RD and RD/PLCδ4 cells indicated that the enhanced expression of PLCδ4 in RD cells increased the phosphorylation of PRAS40 (T246), Chk2 (T68), WNK1 (T60), and Akt (S273) in RD/PLC δ4 cells (Figure 4A,B). Wnk-1 regulates ion transport across cell membranes in mammals. Chk2 activation is essential for cell cycle arrest in G1 and G2/M, and Chk2 is a multiform kinase that phosphorylates several distinct cellular substrates to protect the cell in response to DNA damage. We focused on phospho PRAS40, which was expressed in the RD/PLCδ4 cells at least twice as much as the wild type, and on pAkt, as phospho-PRAS40 is a substrate of Akt. To verify that there were indeed differences in the expression of phosphorylated proteins of the phospho-kinase array, protein immunoblotting was performed. Results for PRAS and Akt phospho-proteins matched the results observed in the array in RD/PLCδ4 cells (Figure 4A–C).

For further analysis and due to the novel finding that PLCδ4 overexpression appeared to be involved in cell cycle arrest, protein levels of cyclin A and cyclin B1 were also examined. In RD/PLCδ4 cells, cyclin A1 levels were unchanged; in contrast, the levels of endogenous cyclin B1, which is a G2/M-phase checkpoint protein, were found to be enhanced. Similarly, p-Akt and p-PRAS40 expression was increased in RD/PLCδ4 cells when compared with parental RD cells. These data highlight that when PLCδ4 is localized in the nucleus, it is able to arrest cell cycle progression by modifying, in part, the expression of cyclin B1 (Figure 4C).

### 3.4. Identification of Cyclin B1 as a Potential Akt Substrate in the Nucleus

**Figure 5:**
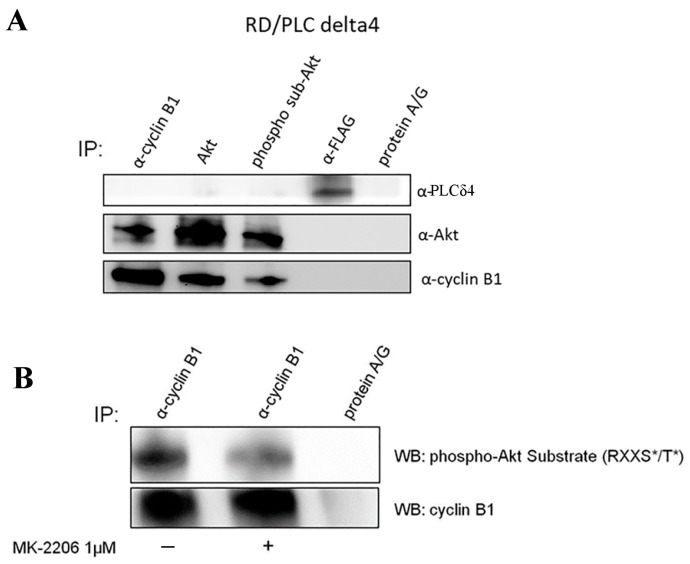
Cyclin B1 is a substrate for Akt in RD/PLCδ4 cells. (**A**) RD/PLCδ4 lysate (1 mg/sample) was either immunoprecipitated with anti-cyclin B1 antibody, anti-Akt antibody, anti–phospho-Akt substrate (K/R-x-K/R-x-x-S*/T*) antibody, anti-FLAG antibody, or protein A:G:sepharose. The blots were probed with the indicated antibodies. (**B**) RD/PLCδ4 cells were treated for 6 h with Akt inhibitor MK-2206, and cell lysates were immunoprecipitated with anti-cyclin B1 antibody. The blot was probed with anti-cyclin B1 antibody and anti–phospho-Akt substrate (K/R-x-K/R-x-x-S*/T*) antibody. Three replicates per tested concentration and at least three independent experiments were performed.

We next evaluated whether cyclin B1 and PLCδ4 were substrates of Akt. Cell lysates of RD/PLCδ4 cells were immunoprecipitated with the anti-phospho-Akt substrate antibody, anti-Akt, anti-cyclin B1, anti-Flag (PLCδ4), or control (Protein A: G-sepharose) and then blotted with anti-PLCδ4, anti-Akt, anti-cyclin B1, and anti-AKT phospho-substrates. Immunoblotting revealed that anti-Akt phospho-substrate and anti-Akt both pulled down cyclin B1 but not PLCδ4, demonstrating that Akt-1 interacts with cyclin B1 and phosphorylates this protein and that PLCδ4 binds neither Akt nor cyclin B1 (Figure 5A). Therefore, PLCδ4 overexpression activates the Akt pathway. In addition, the inhibitor MK2206 was used to further demonstrate that cyclin B1 phosphorylation is Akt-dependent (Figure 5B). 

**Figure 6:**
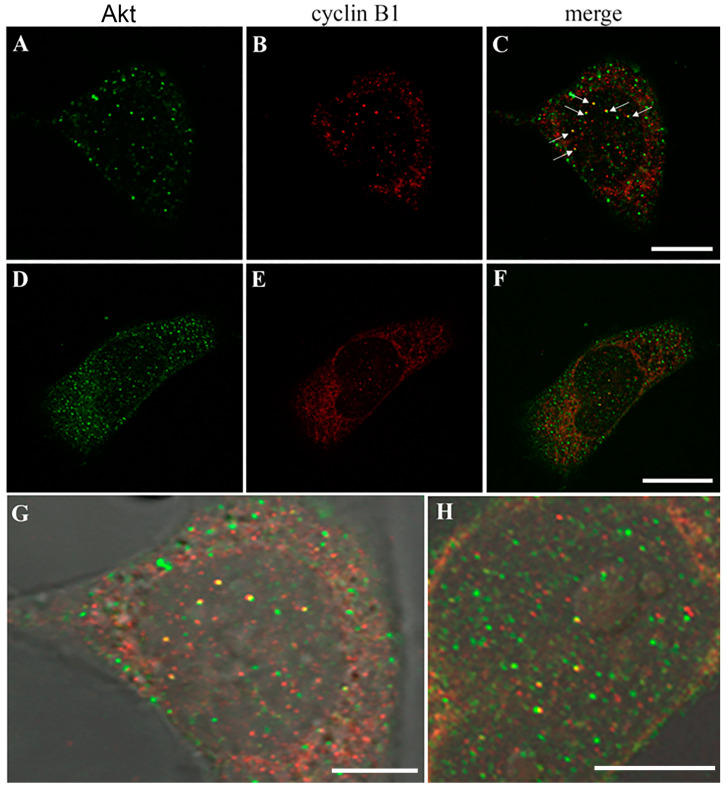
Immunolocalization of Akt (green fluorescence, (**A**,**D**)) and cyclin B1 (red fluorescence; (**B**,**E**)) in RD/PLCδ4 cells. The merge of two proteins appears in (**C**,**F**) and can be better evaluated at the highest magnification (**G**,**H**). (**G**,**H**) micrographs are enlargements of figures (**C**,**F**) and allow us to evidence the presence of yellow staining also localized close to the nucleolus. Bars: 10 µm for (**A**–**F**); 5 µm for (**G**,**H**). Results represent the average of three independent experiments.

To investigate the potential interaction between Akt and cyclin B1, we examined their localization in permeabilized cells using CLSM (Figure 6). Both Akt (Figure 6A,D) and cyclin B1 (Figure 6B,E) localize in both the cytoplasm and the nucleus, evidencing their shuttling between the two compartments, but co-localizing staining can be observed only inside the cell nucleus where yellow, fluorescent dots (Figure 6C,F) resulted from the merging of Akt (green) and cyclin B1 (red). The nuclear co-localization of the two proteins appears better appreciable at a higher magnification (Figure 6G,H), highlighting the presence of yellow staining also localized close to the nucleolus.

### 3.5. PLCδ4 Silencing Affects Cyclin B1 Expression

**Figure 7:**
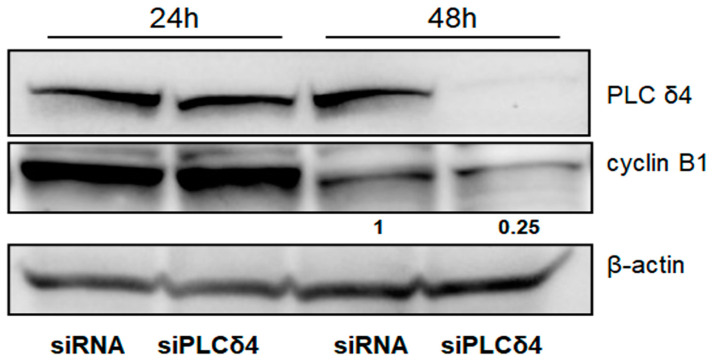
Immunoblotting analysis of A204 cells after silencing of PLC δ4. A total of 30 μg of protein lysates from the indicated cell lines were analyzed for PLC δ4 and cyclin B1 expression by immunoblot. Immunoblot for β-actin was used as loading control. Blots are representative of 2 independent experiments. Densitometric analysis is expressed as arbitrary units.

Since we observed that PLCδ4 is expressed intensely in A204 RMS cells (Figure 1), we knocked down its expression to investigate whether loss of PLCδ4 in these cells also influenced cyclin B1. As expected (Figure 7), there was a significant loss of cyclin B1 protein expression in A204 cells transfected with PLCδ4-siRNA at 48 h compared to the control scrambled siRNA transfected cells, indicating that loss of PLCδ4 has the opposite effect on cyclin B1 in respect to the overexpression experiments on RD cells. These data demonstrate that PLCδ4, localized in the nucleus in ERMS RMS cells, plays a significant role in regulating the expression of cyclin B1.

### 3.6. Effect of Microtubule-Targeted Drugs on RD/PLC δ4

To determine whether PLCδ4 expression influences the sensitivity of RD to stressing agents, the effects of the chemotherapeutic compounds vincristine (Vin) and ixabepilone on RD/PLCδ4 cells were examined. 

The antitumor properties of Vin derive from its ability to inhibit the proliferation of cancer cells, preventing tubulin from polymerizing, thus hindering the formation of microtubules. Ixabepilone binds to beta-tubulin subunits by blocking the mitotic phase of the cell division cycle and inducing cell death. 

To estimate the number of viable cells in multi-well plates, we used the MTT assay as stated in the Materials and Methods. Parental RD cells and the stably transfected RD/PLCδ4 cells were treated for 24, 48, and 72 h with varying serial doses of vincristine or ixabepilone (0.1–10 nM). Overexpression of PLCδ4 caused reduced sensitivity of the cells to vincristine treatment at all tested times.

**Figure 8:**
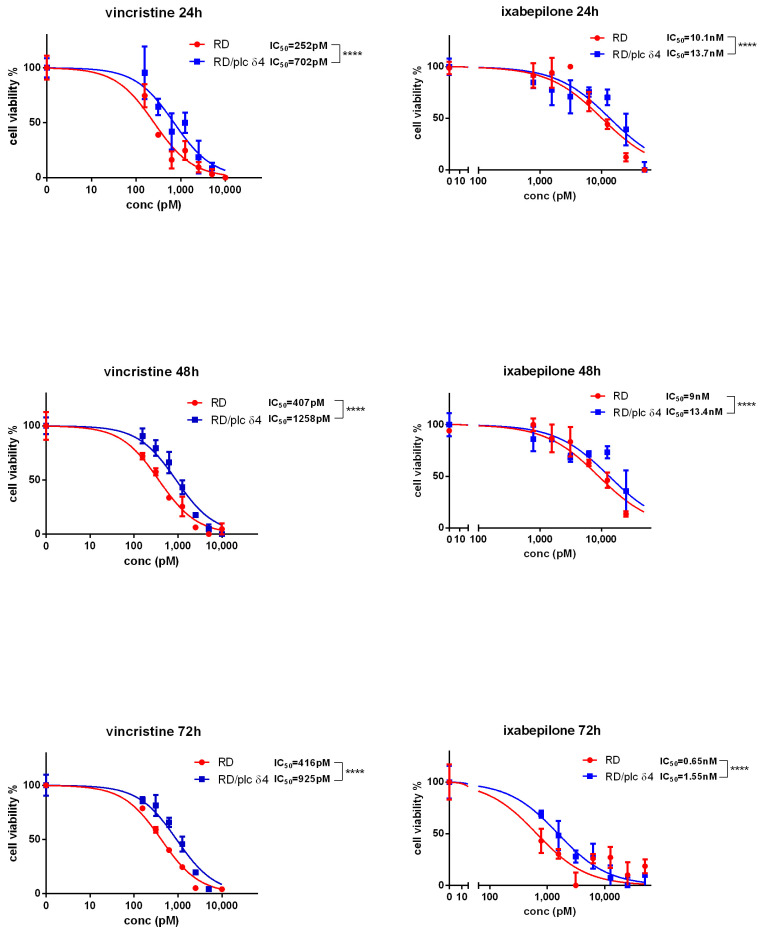
MTT assay in RD and RD/PLCδ4 cells treated with vincristine or ixabepilone. Data were graphed using GraphPad Prism 6 software. **** *p* < 0.0001. Three replicates per tested concentration and at least three independent experiments were performed.

These data suggest once again that PLCδ4 overexpression in RD cells resulted in a significant cell cycle arrest in respect to the parental cells, which consequently promoted reduced sensitivity to microtubule-targeted drugs (Figure 8).

## 4. Discussion

RMS is one of the so-called soft tissue sarcomas, malignant tumors that develop in muscle, fat, or connective tissue. Rhabdomyosarcoma is caused by the transformation of precursors of rhabdomyoblasts, cells that normally give rise to voluntary muscles. The tumor is more common as a sarcoma at a pediatric age and accounts for 3% of pediatric cancers. RMS is a highly malignant tumor characterized by aggressive behavior at the local and systemic levels, with a tendency to metastasize. The therapeutic approach requires surgery, radiotherapy, and multiagent cytotoxic chemotherapy [24]. Considering the ages of the patients and the aggressiveness of the tumor, deepened understanding of the molecular mechanisms driving RMS tumorigenesis is necessary to identify new therapeutic targets. Driven by the finding suggesting a role for nuclear PLCδ4 in cellular proliferation and senescence in mesenchymal stromal stem cells, we examined the PLCδ4 expression profile and regulation in RMS. 

PLCδ4 is described as a phospholipase C enzyme preferentially localized to the nucleus involved in proliferative processes, based upon its initial isolation from regenerating rat liver [16]. More recently, several additional models have supported these findings [25]. 

A large amount of data has shown that polyphosphoinositides are present in the nucleus, where they have a role in cell proliferation and differentiation [26].

Based on previous observations indicating that PLCδ4 is mainly nuclear in mesenchymal stromal stem cells (hASCs) [18], in this investigation, the expression of PLCδ4 was examined in several sarcoma cell lines. PLCδ4 was found to be expressed mainly in the nucleus of A204 ERMS RMS cells and only slightly or was absent in other tested RMS cell lines. A204 is a poorly differentiated embryonal ERMS cell line that carries wild-type p53, whilst each of the other RMS cell lines including the ERMS RD cell line carry mutations in p53. ERMS tumors are more sensitive to chemotherapy and radiation and develop primarily in the head, neck, and genitourinary tract of young children [27]. 

Overexpression of PLCδ4 in RD cells grown under standard conditions was performed to characterize the role of PLCδ4 in RMS. As expected, overexpressed PLCδ4 localized to the nucleus, but interestingly, growth curves of RD cells overexpressing PLCδ4 highlighted an impaired rate of growth as compared to controls (Figure 3C). Moreover, the overexpression of PLCδ4 in RD cells resulted in a significant G2/M arrest with respect to wild-type RD control cells. Studies have shown that G2/M-phase cell cycle arrest presents a major opportunity for inducing cancer cell apoptosis. Morpho-functional analysis showed that RD/PLCδ4 cells became rounded with apoptotic nuclear features, even if, at these experimental times, nuclear fragmentation is not evident. This result was confirmed by an Annexin V-FITC/PI assay. This finding is highly intriguing as, until now, dysregulation/overexpression of PLCδ4 has been proposed to promote oncogenesis through the upregulation of ErbB expression and activation of the ERK pathway [28]. In our experimental model of ERMS RMS using the RD cell line, PLCδ4 appears to exert an opposing effect, which is likely related to the form of cancer, its genetic background, and its origin, in this case, mesenchymal cells. 

To understand how RD cells adapt and respond to changes in their environment when overexpressing PLCδ4, a multiplex antibody array was performed to detect signaling flux through multiple pathways in a single sample. The data showed increased phosphorylation of Akt (S473) and PRAS40 (T246). PRAS40, a substrate of Akt, functions in mTOR signaling and cell growth [29]. 

Flux through PI3K/Akt and its related pathways is in many instances enhanced in tumors and linked to cancer progression [30,31]. Recent studies have highlighted the fact that targeted inhibition of the PI3K/Akt pathway augments cell death in RMS cell lines [32]. We have recently demonstrated that curcumin is able to block the proliferation and viability of RMS cells, blocking them in G2/M in the cell cycle. It also induced apoptotic cell death in three RMS cell lines evaluated (RD, A204, and RH30). In this model, we also observed that curcumin significantly acted on mTOR, more specifically through the inhibition of phosphorylation of AKT on S473 and PRAS40 on S246 (T204) [33]. 

Our study demonstrates that PLCδ4 plays a role in cell cycle control. Overexpression of PLCδ4 in RD cells was significantly associated with the upregulation of cyclin B1, which notably is involved in the inhibition of G2/M cell cycle progression. Moreover, siRNA-mediated knockdown of PLCδ4 in A204 cells led to a reduction in cyclin B1 expression. Consistent with this, findings suggest that nuclear PLCδ4 affects cell cycle progression, in part by affecting the expression of cyclin B1 [17]. Vinca alkaloid agents (vindesine, vinblastine, vinorelbine, vincristine) target microtubule assembly and have important therapeutic effects in special soft tissue sarcoma subtypes, such as RMS and Ewing sarcomas but are known to require cell division to illicit their effects [34]. Proof that the increased expression of PLCδ4 has a role in blocking the cell cycle is the finding that RD/PLCδ4 cells are less sensitive to the action of vincristine. As further proof of this result, we tested ixabepilone’s effect on RD/PLCδ4 cells. Ixabepilone is a well-known drug in the pharmaceutical treatment of cancer. It is a microtubule-stabilizing agent that is mainly used to treat various tumors, especially locally advanced or metastatic breast cancer. Ixabepilone, a derivative of epothilone B, works by attaching to the β-tubulin subunit of microtubules, preventing their disassembly and interfering with the dynamic process essential for cell division [35].

Since our results demonstrated that PLCδ4 overexpression in RD cells enhanced the levels of endogenous cyclin B1, we considered the possibility that PLCδ4 may indirectly induce pAkt1 S473 by regulating the expression of cyclin B1. For the first time, this study demonstrated that that cyclin B1 is complexed with Akt in the nucleus and is phosphorylated by Akt. PLCδ4 binds neither Akt nor cyclin B1. Therefore, PLCδ4 overexpression induces the Akt pathway but in an indirect manner, probably through competition for lipid substrates. Data accumulated over the past 20 years have described the presence of active Akt in the nucleus, where it acts as a crucial factor of nuclear signaling to stimulate survival and differentiation vs. apoptosis in response to growth and differentiating stimuli [36,37]. Previously, the cyclin D1/CDK complex was reported to augment Akt1 activity through phosphorylation of Akt1 at Ser473 [38]. Instead, our study presents evidence demonstrating that cyclin B1 is bound to and phosphorylated by Akt. This study describes a process by which the cell cycle interacts with Akt signaling in a manner that is dissimilar from that reported in several previous studies. In conclusion, our investigation provides novel insights into the role of nuclear PLCδ4.

## 5. Conclusions

These findings collectively establish a groundwork for future studies aiming to unravel the involvement of the PLCδ4/pAkt/cyclin B1 pathway in the RMS pathogenesis, potentially leading to the identification of novel therapeutic targets. All in all, we hypothesize that PLCδ4 could represent a sort of tumor suppressor, given that its forced expression slows growth and reduces sensitivity to vincristine. This could also explain why it seems to be absent in all lines except one. It will be important in the future to evaluate the cellular effects of long-term silencing of PLCδ4 in the A204 line.

## Data Availability

The data presented in this study are available upon request from the corresponding author.

## Supplementary Materials

The following supporting information can be downloaded at https://www.mdpi.com/article/10.3390/biom14091180/s1, Original Western Blots images.
