# Supplementary material for: Phospholipase Cδ-4 (PLCδ4) Acts as a Nuclear Player to Influence Cyclin B Expression in the Embryonal Rhabdomyosarcoma Cell Lines RD and A204
Source: Biomolecules. 2024 Sep 20;14(9):1180. doi: 10.3390/biom14091180 (PMC11430102; doi:10.3390/biom14091180)
Supplement: Supplementary file 1 [file biomolecules-14-01180-s001.zip › biomolecules-3139969-supplementary.pdf]

Fig1

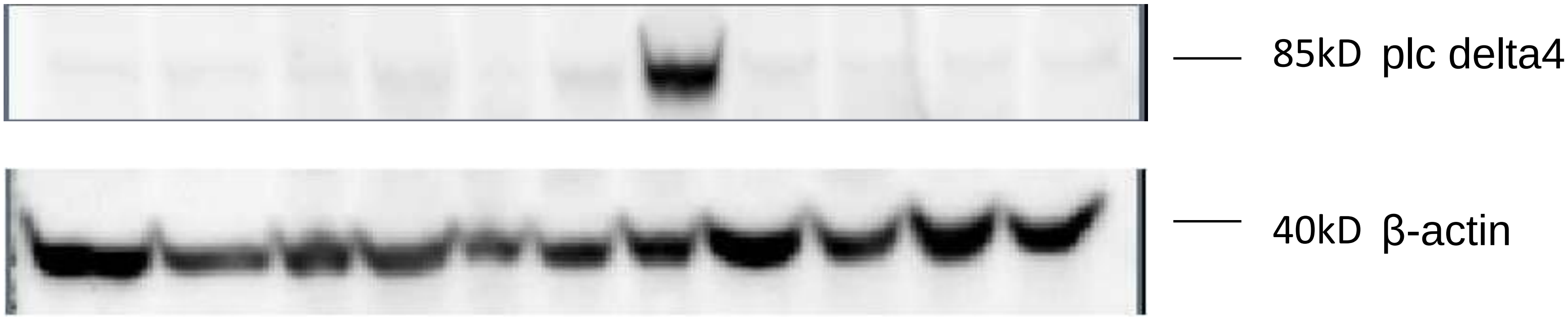

Fig2

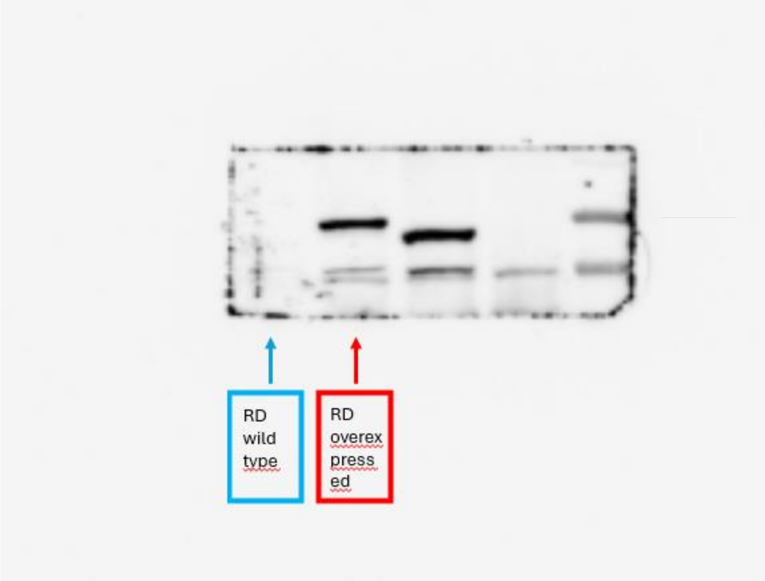

85kD plc delta4

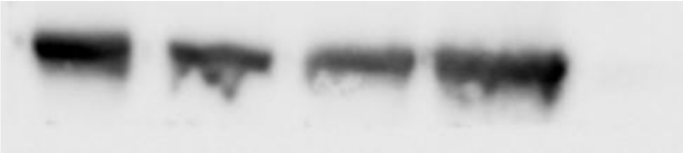

60kD β-tubulin

Fig4 A

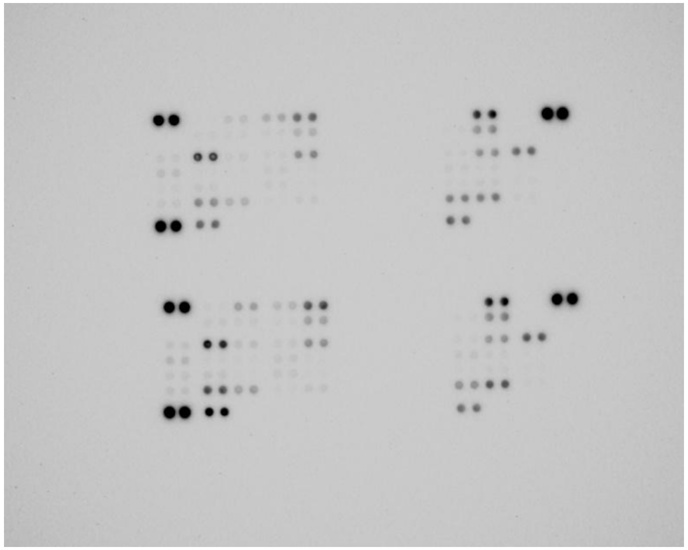

Fig4 C

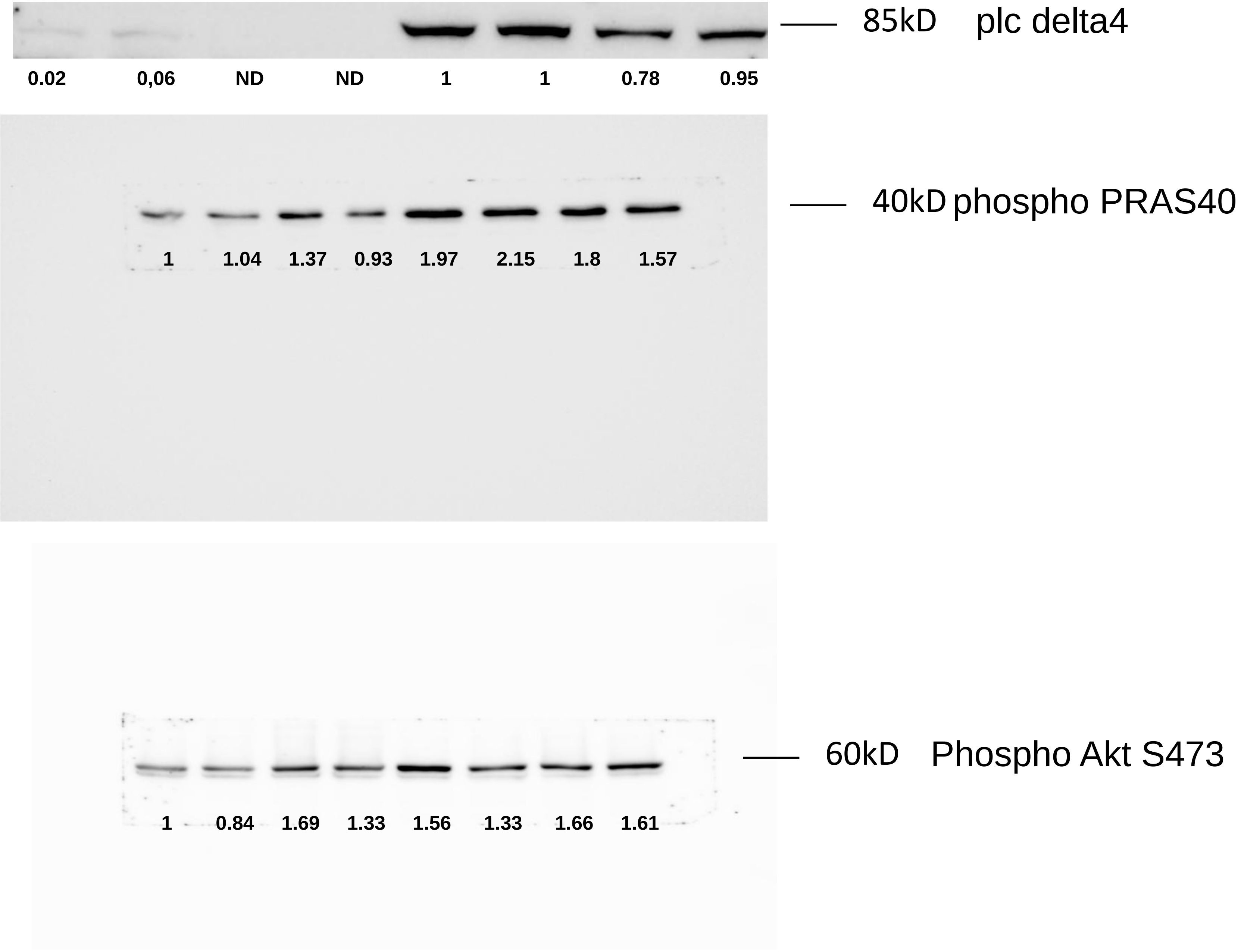

85kD plc delta4
0.02 0,06 ND ND 1 1 0.78 0.95
40kD phospho PRAS40
1 1.04 1.37 0.93 1.97 2.15 1.8 1.57
60kD Phospho Akt S473
1 0.84 1.69 1.33 1.56 1.33 1.66 1.61

Fig4 C

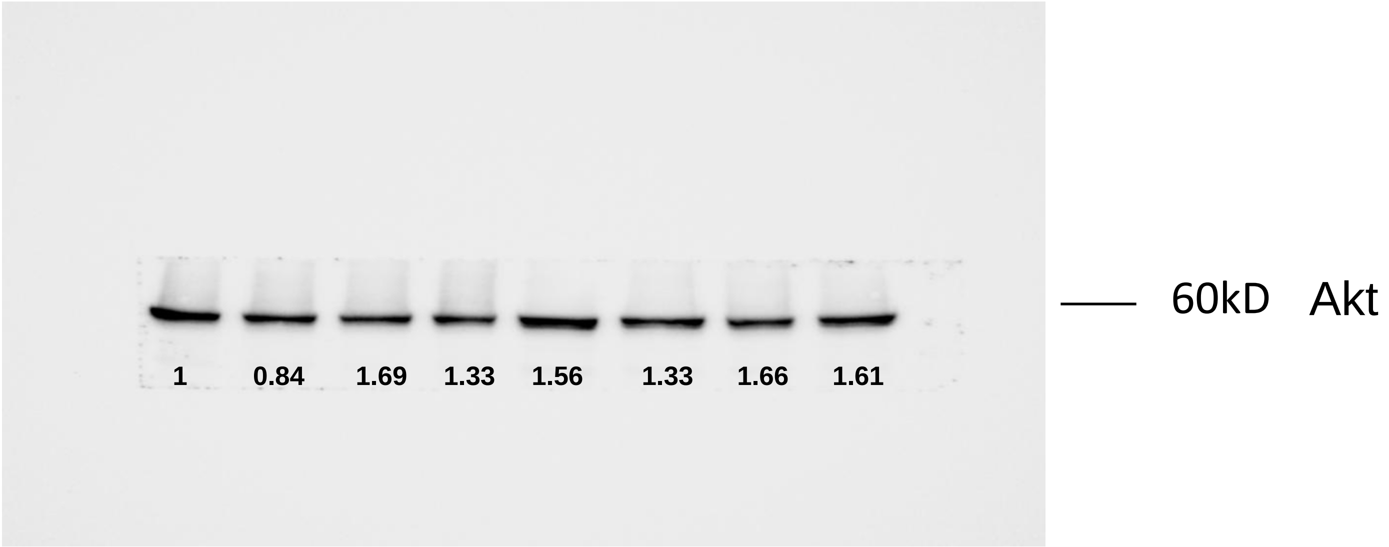
1 0.84 1.69 1.33 1.56 1.33 1.66 1.61
60kD Akt

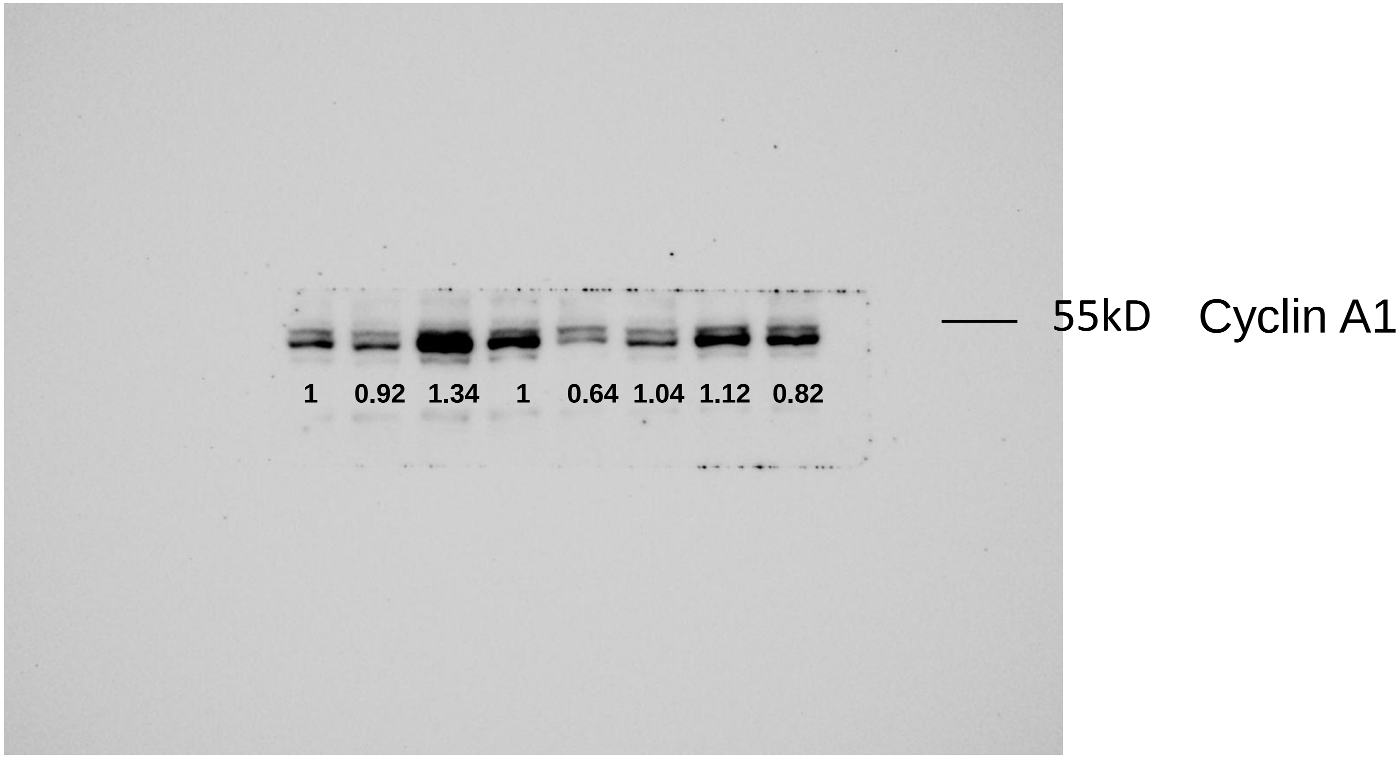
1 0.92 1.34 1 0.64 1.04 1.12 0.82
55kD Cyclin A1

Fig4 C

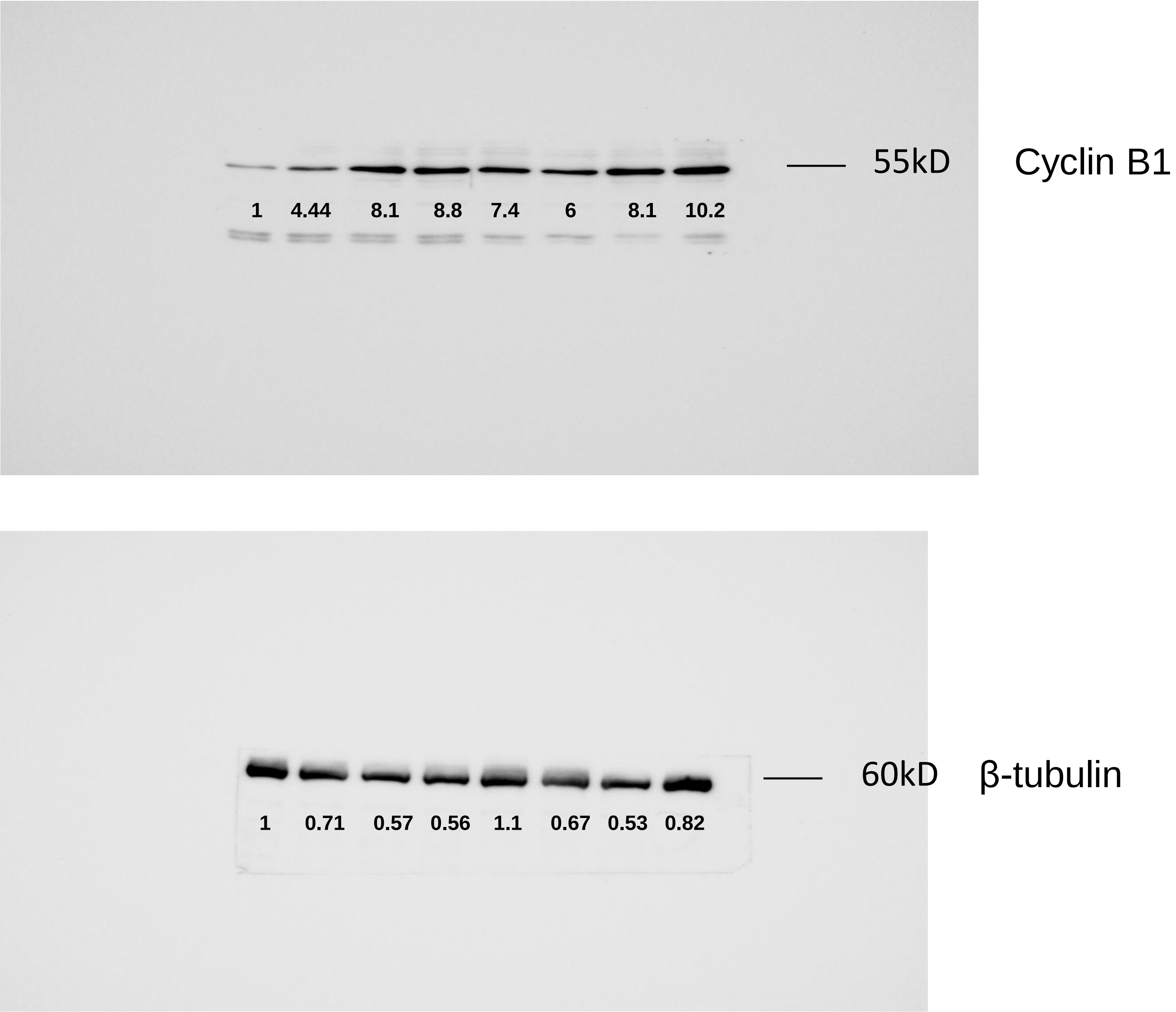
55kD
Cyclin B1
1 4.44 8.1 8.8 7.4 6 8.1 10.2
60kD
β-tubulin
1 0.71 0.57 0.56 1.1 0.67 0.53 0.82

Fig5 A

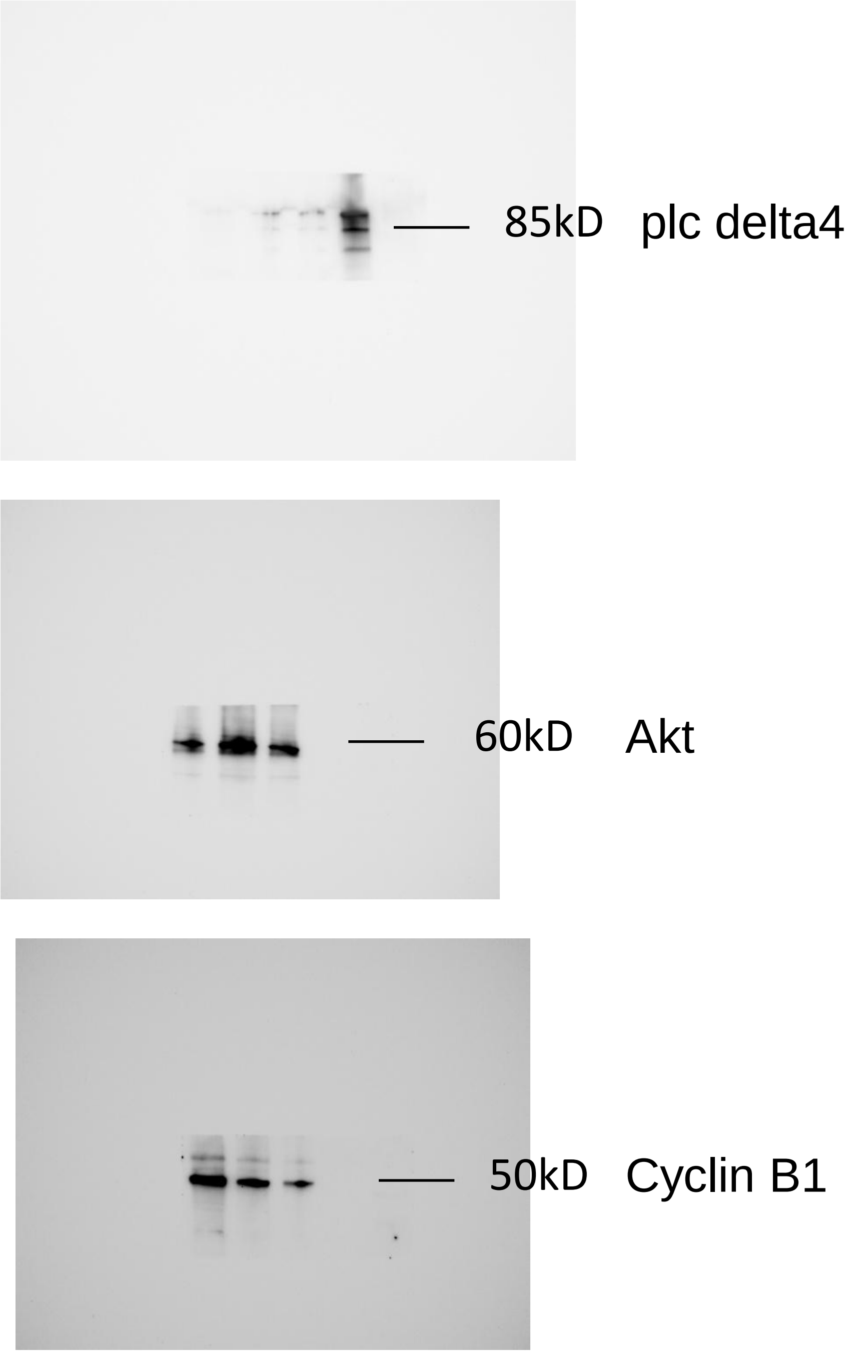

Fig5 B

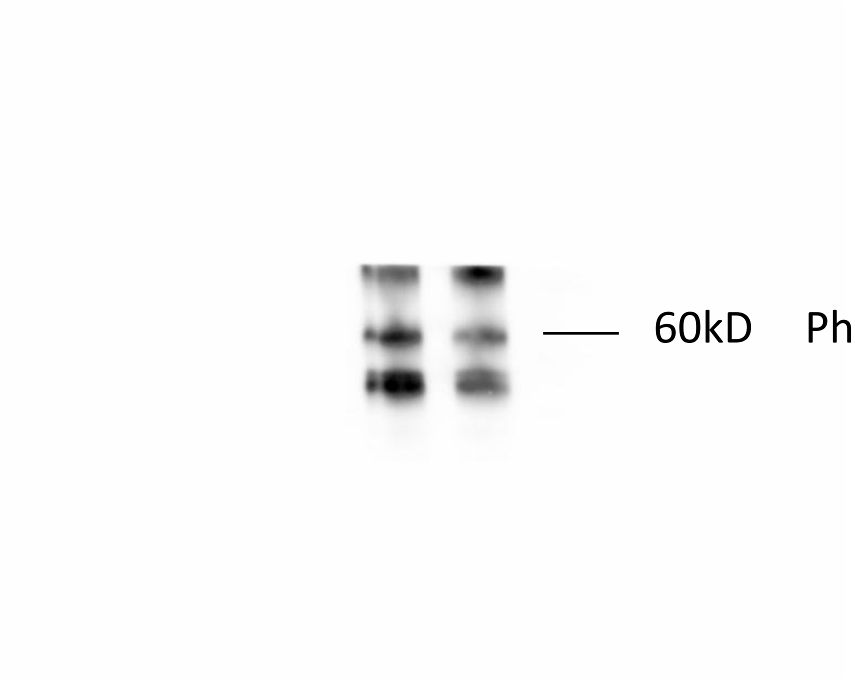

60kD Phospho-Akt substratAkt

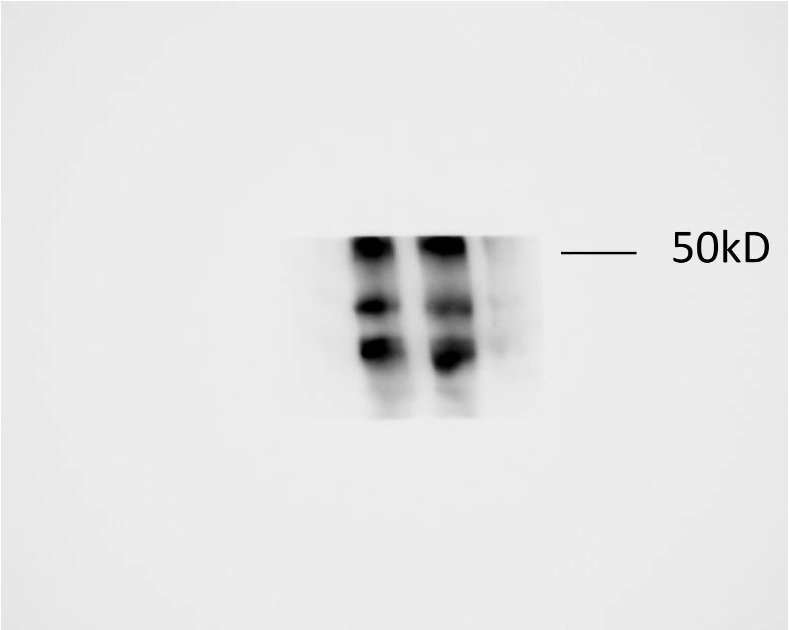

50kD Cyclin B1

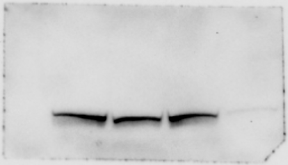

Fig.7 plcdelta 4

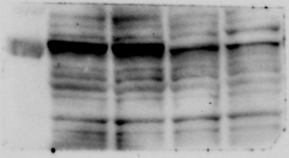

Fig. 7 cyclin B1

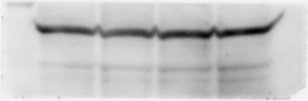

Fig.7 actin
